# Efficient computation of absent words in genomic sequences

**DOI:** 10.1186/1471-2105-9-167

**Published:** 2008-03-26

**Authors:** Julia Herold, Stefan Kurtz, Robert Giegerich

**Affiliations:** 1 Center of Biotechnology, Bielefeld University, Postfach 10 01 31, 33501 Bielefeld, Germany; 2 Center for Bioinformatics, University of Hamburg, Bundesstrasse 43, 20146 Hamburg, Germany

## Abstract

**Background:**

Analysis of sequence composition is a routine task in genome research. Organisms are characterized by their base composition, dinucleotide relative abundance, codon usage, and so on. Unique subsequences are markers of special interest in genome comparison, expression profiling, and genetic engineering. Relative to a random sequence of the same length, unique subsequences are overrepresented in real genomes. Shortest words *absent *from a genome have been addressed in two recent studies.

**Results:**

We describe a new algorithm and software for the computation of absent words. It is more efficient than previous algorithms and easier to use. It directly computes unwords without the need to specify a length estimate. Moreover, it avoids the space requirements of index structures such as suffix trees and suffix arrays. Our implementation is available as an open source package. We compute unwords of human and mouse as well as some other organisms, covering a genome size range from 10^9 ^down to 10^5 ^bp.

**Conclusion:**

The new algorithm computes absent words for the human genome in 10 minutes on standard hardware, using only 2.5 Mb of space. This enables us to perform this type of analysis not only for the largest genomes available so far, but also for the emerging pan- and meta-genome data.

## Background

### Sequence statistics and unique substrings

Word statistics is a traditional field of genome research. For word-length 1, GC-content is a basic characteristic noted for each organism, and dinucleotide relative abundance profiles provide a reliable genomic signature [1]. Dinucleotide content also distinguishes natural RNA from random sequences [2]. Trinucleotide (codon) usage can reliably predict bacterial genes [3] even in the presence of horizontal gene transfer. Short palindromic words mark the characteristic sites of restriction enzymes in bacteria, and are therefore *under *represented in bacterial genomes [4]. A theory of *over*- as well as *under*-represented words has been laid out in [5,6].

*Unique *words are of particular interest. They provide sequence signatures, and microarray probes are often designed to match them. Unique sequences from several genomes exhibiting a perfect match serve as reliable anchors in a multiple genome alignment [7]. Recently, Haubold et al. [8] addressed the problem of efficiently computing shortest unique substrings (using their terminology) in a sequence, and provided a program called SHUSTRING for this purpose. Using this program, they found that there is typically much more unique sequence in a genome than one would expect in a random sequence of the same length. While this observation by itself is not a surprise, given the repetitive nature of genomes, their approach and software allows to quantify this fact. Furthermore, they found unique words to be significantly clustered in upstream regions of genes in human and mouse.

### Absent words

One may take such investigations farther and investigate words that do *not *occur in a genome. We suggest the term "unwords" for shortest words from the underlying alphabet that do not show up in a given sequence.

A first approach at the unwords problem was recently presented by Hampikian and Andersen [9]. Their motivation was to "discover the constraints on natural DNA and protein sequences". However, there is no evidence that such constraints exist. The absence of certain shortest words in a sequence data base, no matter what (finite) size it has, is a mathematical necessity. Speculations about negative selection against certain words have been refuted convincingly in [10]. There, it is shown that human unwords computed in [9] can be explained by a mutational bias rather than negative selection.

Still, there is twofold interest in the capability of efficiently computing unwords.(1) Statistically, it is interesting to see how length and number of unwords in a given genome deviates from expectation in random sequences. (2) Practically, it is useful to know all the unwords when a genome or chromosome is to be extended by insertion of foreign DNA. Combinations of unwords can directly serve as tags that are guaranteed to be unique in the modified DNA sequence.

### Software for unwords computation

Unfortunately, the software presented in [9] is slow and difficult to use: It reads Genbank files rather than the more space efficient Fasta format – and space matters a lot when dealing with genomes as large as human and mouse. It runs an internal conversion routine for over 50 minutes before starting unwords computation. The program generates an excessive number of files that may break your file systems. The C code is platform dependent and internal constants must be adapted. Finally, the human unwords data computed with the program according to [9] appear to be incomplete (and hence incorrect).

In order to make unwords computation possible in an efficient and reliable way, we present here a new algorithm and the software implementing it. Efficient computation of unwords can be done from an index data structure such as a suffix tree or an (enhanced) suffix array [11]. For example, in [8] a suffix tree was used to compute unique substrings. In fact, our first unwords-program was an extension to the VMATCH software [12], which is based on enhanced suffix arrays. However, index data structures must be built in memory and are space-consuming. Hence, we developed a direct approach that works more efficiently, because the overall sequence need not be kept in main memory. Computing the unwords of the human genome, for example, takes about 10 minutes computation time on a Linux PC with a single 2.4 MHz CPU. The space requirement is 2.5 megabytes.

In this article, we describe the new program UNWORDS and report its application to the genomes of human, mouse, and other organisms, covering a genome size range from 10^9 ^down to 10^5 ^bp.

## Results

### Problem statement

Let Σ be a finite alphabet of at least two letters. Let |Σ| denote the cardinality of Σ. In genome analysis, Σ = {a, c, g, t} and |Σ| = 4. A word is a sequence of letters from the alphabet. The terms "word" and "sequence" are equivalent, but are used here to indicate that a word is short and a sequence is long. |*w*| denotes the length of a word. If |*w*| = *q*, we speak of a *q*-word.

A word *w *over Σ is an *unword *of a sequence *G *if (1) it does not occur as a substring of *G*, and (2) all words over Σ shorter than *w *do occur in *G*. Note that the unword length is uniquely defined for a given genome *G*.

The built-in minimality requirement in this definition is motivated by the fact that when *w *is an unword of length *q *in *G*, it has 2|Σ| one-letter extensions that also do not occur in *G*. Therefore, asking for missing words longer than *q *would introduce a substantial proportion of redundant results.

Similar to shortest unique substrings, the length of unwords is expected to increase with genome size. For fixed unword length, the number of unwords is expected to decrease while |*G*| increases. Given *G*, let *q *be the unword length. It is easy to see that 1 ≤ *q*. To derive an upper bound on *q*, let *w *be a shortest unique substring in *G *and let ℓ = |*w*|. Consider the following cases:

• If |*w*| = |*G*|, then for any *a *∈ Σ, *wa *is an unword. Hence *q *≤ |*wa*| = ℓ + 1.

• If |*w*| < |*G*| and *w *is not a suffix of *G*, then *wa *occurs in *G *for exactly one letter *a*. Hence *wb *for any *b *∈ Σ\{*a*} is an unword. This implies *q *≤ |*wb*| = ℓ + 1.

• If |*w*| < |*G*| and *w *is not a prefix of *G*, then aw occurs in *G *for exactly one letter *a*. Hence *bw *for any *b *∈ Σ\{*a*} is an unword. This implies *q *≤ |*wb*| = ℓ + 1.

Thus we conclude 1 ≤ *q *≤ ℓ + 1.

The problem of *unword analysis *of a given sequence *G *(typically a complete genome) is to determine all unwords of *G*. The double-stranded nature of DNA lets unwords always show up in complementary pairs, as each word present implies the presence of its Watson-Crick complement on the opposite strand. Sometimes, however, an unword is self-complementary, and hence a "pair" represents only a single word. Therefore, we report unword numbers rather than numbers of pairs (in contrast to [8]).

Computation of *q*-word statistics for small *q *is straightforward. Efficient computation of unwords when *q *is unknown, however, requires more advanced techniques. Our unword analysis algorithm is described in the section on computational methods.

### Unword statistics

The unword analysis problem is mathematically well defined. Unwords must exist for any sequence. The interesting question is their size and number, compared to what one would expect given the alphabet size and the length of *G*.

Let *w *be a word of length |*w*|, *w *[*i*] the *i*-th letter in *w*, *G *a genomic sequence and ℙ[*w *[*i*]] the relative frequency of nucleotide *w *[*i*] in *G*. The probability for *w *to occur by chance (i.e. at a fixed position in a random sequence *s *of the same composition and length as *G*) is then $\mathbb{P}[w]=\prod_{i=1}^{\left|w\right|}\mathbb{P}[w[i]]$. The expectation value for (the number of occurrences of) *w *in *s *is E[*w in s*] ≈ ℙ[*w*]·|*G*|.

Calculating the probability for a word *not *to occur in a specific sequence is quite difficult and not much literature is available. Following Rahmann et al. [13], a good approximation of the probability can be given using the expectation value. A Poisson Distribution is expected for word counts in a genomic sequence, which is $\mathbb{P}[X_{w}=k]=\frac{\lambda {\left(w\right)}^{k}}{k!}\cdot e^{-\lambda (w)}$ with *λ*(*w*) = E[*w in s*], and *k *the number of occurrences of the word *w*. Now let *k *= 0. Then

(1) $$\mathbb{P}[X_{w}=0]=1\cdot e^{-\lambda (w)}$$

The expected number *N *of *q*-words that do not occur is therefore

(2) *N *≈ |Σ|^*q*^*e*^-*λ*(*w*)^

As an example, for a random sequence *G *of length 3.1·10^9 ^and an unword *w *of length 14 and typical composition, we obtain a probability of 1.40082·10^-5 ^for *w *not occurring in *G*. Still, the expected number of unwords of length 14 is 2590.798, while for length 13, it is only 5.823108·10^-13^. For even shorter unwords, it is practically zero.

### Unwords algorithm

For convenience, we map each of the four letters of the DNA-alphabet to an integer in the range 0 to 3 as follows: *ā *= 0, $\bar{c}$ = 1, $\bar{g}$ = 2, $\bar{t}$ = 3. Moreover, for any fixed value *q*, we use a standard method to map each possible *q*-word to a number in the range [0, 4^*q *^- 1]. That is, we define $\phi_{q}(w)=\sum_{i=1}^{q}\bar{w[i]}\cdot 4^{q-i}$ for any *q*-word *w*. In other words, *q*-words are mapped to their rank in the corresponding lexicographic order. Substrings in *G *containing at least one wildcard (e.g. N) are ignored. The integer value *φ*_*q *_(*w*) serves as an index into a bit table Ω_*q *_such that for all sequences *w *of length *q *we have: Ω_*q *_[*φ*_*q *_(*w*)] = 1 if and only if *w *occurs as a substring in the genome *G*. Let |Ω_*q*_| denote the number of 1-entries in Ω_*q*_.

Initially we set all bits in Ω_*q *_to 0. This requires $O\left(\frac{4^{q}}{\omega }\right)$ time, where *w *is the computer word size. Then we sweep a window of width *q *over *G *from left to right. For the first window *G *[1..*q*] we determine the integer code *φ*_*q *_(*G *[1..*q*]) as defined above in *O*(*q*) time. For each of the remaining *n *- *q *windows, say at start position *i *+ 1, we compute *φ*_*q *_(*G*[*i *+ 1..*i *+ *q*]) in constant time from *φ*_*q *_(*G*[*i..i *+ *q *- 1]) according to the following equation:

(3) $$\phi_{q}(G[i+1 ..i+q])=(\phi_{q}(G[i..i+q-1])-4^{q-1}\cdot \bar{G[i]})\cdot 4+\bar{G[i+q]}$$

Thus the computation of the *n *- *q *+ 1 integer code requires *O*(*n*) time. The multiplication and addition in can be implemented by fast bit-shift and bit-or operations. If *j *is the current integer code and Ω_*q *_[*j*] is 0, then we set Ω_*q *_[*j*] to 1 and increment a counter of the number of 1-entries in Ω_*q*_. This can be done in constant time. Note that once |Ω_*q*_| = 4^*q*^, we can stop scanning *G*. While the time requirement of this algorithm is $O\left(n+\frac{4^{q}}{\omega }\right)$ it uses *O*(1) + 2*q *+ 4^*q *^bits of space, as only *q *consecutive letters in *G *need to be stored in memory.

If |Ω_*q*_| = 4^*q*^, i.e. all 4^*q *^entries in Ω_*q *_are 1, then we know that all possible *q*-words occur in *G*. Hence there is no unword of length *q *in *G*. On the other hand, if after processing all *q*-words in *G*, |Ω_*q*_| < 4^*q*^, there are some unwords of length *q*. If additionally |Ω_*q*-1_| = 4^*q*-1^, then we know that *q *is the smallest value such that unwords of length *q *exist. The unwords can easily be computed by determining all *j *such that Ω_*q *_[*j*] = 0. Given *j*, one determines the corresponding *q*-word *w *satisfying *φ*_*q *_(*w*) = *j *in *O*(*q*) time. Thus the unwords are enumerated in *O*(4^1 ^+ *qz*) time where *z *is the number of unwords.

Let q* be the smallest value such that there are unwords of length q*. Consider the possible range of values for *q *for a given genome length *n*. Let *q*^max ^= ⌈log_4 _(*n *+ 1)⌉. Then $4^{q^{\max}}=4^{\left\lceil \log_{4}(n+1)\right\rceil }\geq n+1>n\geq n-q^{\max}+1$. Note that *G *contains *n *- *q*^max ^+ 1 substrings of length *q*^max^. Hence *G *is too short to accommodate all possible *q*^max^-words and therefore there are some unwords of length *q*^max^. Thus q* ≤ *q*^max^, i.e. we can restrict the search for q* to the range [1, *q*^max^].

There are basically two strategies to determine q*. The first strategy (linear search) starts with *q *= 1 and increments *q *until |Ω_*q*_| < 4^*q*^. Then q* = *q*. The space requirement is *O*(1) + 2q* + 4^q^* and the running time is

(4) $$O(4^{q^{*}}+q^{*}z)+\sum_{q=1}^{q^{*}}O\left(n+\frac{4^{q}}{\omega }\right)=O(4^{q^{*}}+q^{*}z)+O(q^{*}n)+O\left(\frac{4^{q^{*}+1}}{\omega }\right),$$

where *z *is the number of unwords. Note that we have $n\geq 4^{q^{*}-1}=\frac{4^{q^{*}+1}}{4^{2}}\geq \frac{4^{q^{*}+1}}{\omega }$ under the realistic assumption that the machine word size *ω *is at least 4^2^. Hence *n *dominates the last term in (4). Thus the overall running time for the linear search is *O*(4^q^* + q* (*n *+ *z*)).

The second strategy determines q* by a binary search in the range [1, *q*^max^], as described in Table 1. The strategy is optimal in the sense that it tests a minimal number of possible values of *q *before it arrives at q*. Unfortunately, a value *q' *determined in line 8 of Table 1, may or may not be modified later in the loop, which means that one has to store the corresponding table Ω_*q' *_or recompute it later. The running time of the binary search is $O(4^{q^{*}}+q^{*}z)+\log_{2}q^{\max}(n+\frac{4^{q^{\max}-1}}{\omega })$. Its space requirement is *O*(1) + 2*q*^max ^+ $4^{q^{\max}}$.

**Table 1:** Algorithm for computing q* by a binary search strategy.

1: determine sequence length *n*
2: *l *← 1
3: *r *← ⅸlog_4 _(*n *+ 1)ⅹ
4: **while ***l *≤ *r ***do**
5: *q *← (*l *+ *r*)/2
6: compute Ω_*q*_
7: **if **|Ω_*q*_| < 4^*q *^**then**
8: *q' *← *q*
9: Ω_*q' *_← Ω_*q*_
10: *r *← *q *- 1
11: **else**
12: *l *← *q *+ 1
13: **end if**
14: **end while**
15: q* ← *q'*
16: Ω_q* _← Ω_*q'*_
17: **for all ***j *∈ [0, 4^q^* - 1] **do**
18: **if **Ω_q* _[*j*] = 0 **then**
19: print *w *such that *φ*_q* _(*w*) = *j*
20: **end if**
21: **end for**

### Testing

We used our first implementation (based on suffix-arrays) of an unwords algorithm to cross-validate the program presented here. Applied to the human genome, both algorithms (which are completely independent) produce the same set of unwords. This makes us sure that our set of 104 human unwords is indeed complete, in contrast to the 80 unwords reported in [9]. (If a smaller genome assembly or repeat masked sequences were used in this earlier study, more rather than less unwords should have been detected.) We computed unwords for six eucaryotic genomes: *Homo sapiens*, Release NCBI36 [14], *Mus musculus*, Release NCBIm36 [15], *Drosophila melanogaster*, Release 5.1 [16], *Caenorhabditis elegans*, Release WS170 [17], *Neurospora crassa *[18] and *Saccharomyces cerevisiae*, Release SGD1.01 [19], including nonchromosomal sequences which could not be assigned to a chromosome. Additionally, unwords for two bacterial genomes were calculated: *Staphylococcus aureus subsp. aureus *strain MSSA476, Refseq number NC_002953 and *Mycoplasma genitalium*, Refseq number NC_000908, as well as for two Archaea genomes:

*Thermococcus kodakarensis*, Release KOD1 [20] and *Methanocaldococcus jannaschii*, Release DSM 2661 [21]. Table 2 gives a summary of genome sizes and unword lengths and numbers. In Table 3, we show the unwords computed from the human genome. We also indicate the number of occurrences expected for each unword – if the genome was a random sequence, which of course is not the case. Deviation of GC content in unwords is summarized in Table 4. Unwords for the other genomes are given in Tables 5, 6, 7, 8, 9, 10, 11, 12.

**Table 2:** Genome sizes (including sequences not assigned to a chromosome), the logarithm of the genome size to the base of 10, length and number of unwords of the analyzed genomes

Organism	Genome size	⌊log_10 _|*G*|⌋	⌊log_4 _|*G*|⌋	#unwords	length
*H. sapiens*	≈ 3.1 Gb	9	15.8	104	11
*M. musculus*	≈ 2.7 Gb	9	15.7	192	11
*D. melanogaster*	≈ 132 Mb	8	13.5	104	11
*C. elegans*	≈ 100 Mb	8	13.3	2	10
*N. crassa*	≈ 34 Mb	7	12.5	2262	11
*S. cerevisiae*	≈ 12 Mb	7	11.8	4	9
*S. aureus*	≈ 2.79 Mb	6	10.7	248	8
*T. kodakarensis*	≈ 2.08 Mb	6	10.5	1	8
*M. jannaschii*	≈ 1.66 Mb	6	10.3	3	6
*M. genitalium*	≈ 0.58 Mb	5	9.6	5	6

**Table 3:** Unwords for the human genome and their expected number of occurrences. The four words which are also unwords for the mouse genome are shown in a box.

accgatacgcg	153	accgttcgtcg	153	acgaccgttcg	153	acgatcgtcgg	153
acgcgcgatat	221	acggtacgtcg	153	agcgtcgtacg	153	atatcgcgcgg	153
atatcgcgcgt	221	atcgtcgacga	221	atgtcgcgcga	153	catatcgcgcg	153
ccgaatacgcg	153	ccgacgatcga	153	ccgacgatcgt	153	ccgatacgtcg	153
ccgcgcgatat	153	ccgtcgaacgc	106	ccgttacgtcg	153	cgaacggtcgt	153
cgaatcgacga	221	cgaatcgcgta	221	cgaccgatacg	153	cgacgaacgag	153
cgacgaacggt	153	**cgacgcgatac**	153	cgacgcgtata	221	cgacggacgta	153
cgacgtaacgg	153	cgacgtaccgt	153	cgacgtatcgg	153	cgatcgtgcga	153
cgattacgcga	221	cgattcggcga	153	cgcgacgcata	153	cgcgacgttaa	221
cgcgcataata	319	cgcgcgatatg	153	cgcgctatacg	153	cgcgtaacgcg	106
cgcgtaatacg	221	cgcgtaatcga	221	cgcgtatcggt	153	cgcgtattcgg	153
cgcgttacgcg	106	**cgctcgacgta**	153	cggtcgtacga	153	cgtacgaaacg	221
cgtacgacgct	153	cgtatacgcga	221	cgtatagcgcg	153	cgtatcggtcg	153
cgtattacgcg	221	cgtcgactatc	221	cgtcgctcgaa	153	cgtcgttcgac	153
cgttacgcgtc	153	cgtttcgtacg	222	ctacgcgtcga	153	ctcgttcgtcg	153
gacgcgtaacg	153	gatagtcgacg	221	gcgcgacgtta	153	gcgcgtaccga	106
gcgttcgacgg	106	ggtacgcgtaa	221	**gtatcgcgtcg**	153	gtccgagcgta	153
gtcgaacgacg	153	taacgtcgcgc	153	tacgcgattcg	221	tacgcgcgaca	153
tacgctcggac	153	tacggtcgcga	153	tacgtccgtcg	153	**tacgtcgagcg**	153
tagcgtaccga	221	tatacgcgtcg	221	tatcgcgtcga	221	tatgcgtcgcg	153
tattatgcgcg	321	tattcgcgcga	221	tcgacgcgata	221	tcgacgcgtag	153
tcgatcgtcgg	153	tcgattacgcg	221	tcgcacgatcg	153	tcgccgaatcg	153
tcgcgaccgta	153	tcgcgacgtaa	221	tcgcgcgaata	221	tcgcgcgacat	153
tcgcgtaatcg	221	tcgcgtatacg	221	tcggtacgcgc	106	tcggtacgcta	221
tcgtacgaccg	153	tcgtcgacgat	221	tcgtcgattcg	222	tgtcgcgcgta	153
ttaacgtcgcg	221	ttacgcgtacc	221	ttacgtcgcga	221	ttcgagcgacg	153

**Table 4:** GC content of Human, Mouse, *Drosophila melanogaster*, *Caenorhabditis elegans*, *Saccharomyces cerevisiae*, *Staphylococcus aureus *and *Mycoplasma genitalium *as well as the GC content of the associated unwords.

Organism	Genome GC%	Unword GC%
*H. sapiens*	≈ 38	≈ 45–72
*M. musculus*	≈ 40	≈ 54–72
*D. melanogaster*	≈ 40	≈ 45–90
*C. elegans*	≈ 35	≈ 80
*S. cerevisiae*	≈ 38	≈ 89–100
*S. aureus*	≈ 33	≈ 50–100
*M. genitalium*	≈ 32	≈ 66–100

**Table 5:** Unwords for the Mouse genome.

aacgcgtatcg	aatcgcgcgat	acccgcgtacg	accgcgatacg	acgaacgtcga	acgacgcgata
acgacgtacgg	acgattcgacg	acgattcgcgt	acgcgaaacga	acgcgaatcgt	acgcgtcgaaa
acgcgtcgcga	acgcgtcgcta	acggtcgtcga	acgttcgaacg	acgttcgaccg	actcgtcgcga
atcgacgcgcg	atcgcgcgatt	atcgcggtacg	atcgtaccgcg	atcgtacgccg	atcgtcgaccg
attacgcgcga	attacgcgcgg	attacgtcgcg	attcgcgcgta	attgcgtcgcg	cccgatacgcg
ccgatacgcgc	ccgcgatacga	ccgcgcgataa	ccgcgcgtaat	ccgcgcgtata	ccggtcgtacg
ccgtacgtcgt	ccgtcgaatcg	cgaatttcgcg	cgacgagcgta	cgacgcgataa	cgacgcgatac
cgacgcgtaac	cgacggatacg	cgacgtaacgc	cgacgttaacg	cgactaacgcg	cgatacgacga
cgatacgccga	cgatacgcgtt	cgatagtcgcg	cgatcgacgcg	cgatcgcgtaa	cgatcgtacga
cgatcgtcgca	cgattcgacgg	cgattgacgcg	cgcatatcgcg	cgccgattacg	cgcgaaattcg
cgcgaccgata	cgcgacgcaat	cgcgacgtaat	cgcgactatcg	cgcgatacgaa	cgcgatacgac
cgcgatatcac	cgcgatatccg	cgcgatatgcg	cgcgatcggta	cgcgcgtaacg	cgcgcgtcgat
cgcggtacgat	cgcgtaacgta	cgcgtatcggg	cgcgtcaatcg	cgcgtcacgta	cgcgtcgatcg
cgcgtcgatta	cgcgttagtcg	cgctcgacgta	cggacgtcgta	cggatatcgcg	cggcgtacgat
cggcgtcgtaa	cgggcgtaacg	cggtcgaacgt	cggtcgacgat	cgtaatcgcga	cgtaatcggcg
cgtaccgcgat	cgtacgaccgg	cgtacgatcgc	cgtacgcgggt	cgtatccgtcg	cgtatcgcgag
cgtatcgcggt	cgtccgatcga	cgtcgaatcgt	cgtcgacgagc	cgtcgcgttaa	cgtcgcgttag
cgtcgttacgc	cgttaacgtcg	cgttacgcccg	cgttacgcgcg	cgttcgaacgt	cgttcgaccga
cgttgcgcgaa	cgttgcgtcga	ctaacgcgacg	ctcgcgatacg	ctcgcgtacga	gcgatcgtacg
gcgcgatacga	gcgcgtacgac	gcgcgtatcgg	gcgtaacgacg	gcgttacgtcg	gctcgtcgacg
gtatcgcgtcg	gtcgcgaacta	gtcgcgcgata	gtcgtacgcga	gtcgtacgcgc	gtcgtatcgcg
gtgatatcgcg	gttacgcgtcg	taaccgcgcga	taatcgacgcg	taccgatcgcg	tacgacgtccg
tacgcgcgaat	tacgctcgtcg	tacggacgcga	tacgtcgagcg	tacgtgacgcg	tacgttacgcg
tagcgacgcgt	tagttcgcgac	tatacgcgcgg	tatcgcgcgaa	tatcgcgcgac	tatcgcgtcgt
tatcggcgcga	tatcggtcgcg	tcatcgcgcga	tcgacgaccgt	tcgacgcaacg	tcgacgcgtaa
tcgacgttcgt	tcgatcggacg	tcgcgacgaaa	tcgcgacgagt	tcgcgacgcgt	tcgcgattacg
tcgcgccgata	tcgcgcgatga	tcgcgcggtta	tcgcgcgtaat	tcgcgtaccga	tcgcgtacgaa
tcgcgtacgac	tcgcgtccgta	tcggcgtatcg	tcggtacgcga	tcggtcgaacg	tcgtacgatcg
tcgtacgcgag	tcgtatcgcgc	tcgtatcgcgg	tcgtcgaacga	tcgtcgtatcg	tcgttcgacga
tcgtttcgcgt	tgcgacgatcg	ttaacgcgacg	ttacgacgccg	ttacgcgatcg	ttacgcgcgaa
ttacgcgtcga	ttatcgcgcgg	ttatcgcgtcg	ttcgcgcaacg	ttcgcgcgata	ttcgcgcgtaa
ttcgtacgcga	ttcgtatcgcg	tttcgacgcgt	tttcgtcgcga		

**Table 6:** Unwords for the *C. elegans *genome.

acccccccag	ctgggggggt

**Table 7:** Unwords for the *D. melanogaster *genome.

acccctaggga	acccctctacg	acccggtaggg	accctaccggg
acctagcgcgc	acctagcgcgt	acctagcgtga	acctaggtctg
acgcgctaggt	acggccgtacc	acgggaggttc	acgtcccgcta
actaggtaccg	aggcccgcgcg	aggcccgctat	agggtacgccg
agtataggccg	atagcgggcct	cacgcgtgggg	cagacctaggt
ccccacgcgtg	ccccggcctag	ccccgtagggc	cccgcgttaag
cccggtagggt	cccggtctagg	cccgtacgcgc	ccctaccgggt
ccctacggggc	ccctaggcacg	ccggtagctag	ccggtagggta
cctacgcgtca	cctacgtagag	cctagaccggg	cctagggtccg
cctataggccg	cgcgcgggcct	cgcgctagcgc	cgcgctaggcc
cgcggggtacc	cgcgtagtcta	cgctagggccg	cggaccctagg
cggccctagcg	cggcctatact	cggcctatagg	cggcgtaccct
cggggcccgac	cgggtagactc	cgggtcgctag	cggtacctagt
cggtcctatcc	cgtagaggggt	cgtccgtagca	cgtgagggacc
cgtgcctaggg	ctagcgacccg	ctagctaccgg	ctaggccgggg
ctctacgtagg	cttaacgcggg	gaacctcccgt	gacctactaga
gacctaggtac	gacgctagggc	gagtctacccg	gccccgtaggg
gccctacgggg	gccctagcgtc	gcgcgctaggt	gcgcgtacccc
gcgcgtacggg	gcgctagcgcg	gcggccctacc	gcgggtacccc
gctagggtacc	ggataggaccg	ggcctagcgcg	gggacgttaga
ggggtacccgc	ggggtacgcgc	ggtaccccgcg	ggtaccctagc
ggtacggccgt	ggtagggccgc	ggtccctcacg	ggtccgcgcta
gtaacgcggac	gtacctaggtc	gtccgcgttac	gtcgggccccg
gtcggtcccta	taccctaccgg	tagactacgcg	tagcgcggacc
tagcgggacgt	tagggaccgac	tcacgctaggt	tccctaggggt
tctaacgtccc	tctagtaggtc	tgacgcgtagg	tgctacggacg

**Table 8:** Unwords for the *S. cerevisiae *genome.

ccccgggga	cgccccccg	cggggggcg	tccccgggg

**Table 9:** Unwords for the *S. aureus *genome (strain MSSA476).

aacccccc	acacgggg	accccgcg	acccgggc	acccgggg	accggcgg
acgccggg	acgcgggc	acggcccg	acgggacc	acgggccc	acgggggg
actccggg	actcgggc	agcccggg	agccgagg	aggccccc	aggccccg
aggcccgg	aggggggg	atccgggg	cacggaga	cacggggc	cacggggg
cagcgggg	caggccgc	caggccgg	cagggccg	ccacggag	cccacgga
cccagggg	cccccccc	ccccccct	ccccccgc	ccccccgt	cccccggg
cccccgtg	ccccgagg	ccccgcgc	ccccgctg	ccccggag	ccccggat
ccccggcc	ccccggcg	ccccgggc	ccccgggt	ccccgtgt	cccctggg
cccgaggg	cccgcagg	cccgcggg	cccggagc	cccggagt	cccggcgt
cccgggag	cccgggcc	cccgggct	cccggggg	ccctaggg	ccctccgc
ccctcggg	ccgagagc	ccgccccg	ccgccggt	ccgcgccc	ccgcgcgg
ccgcgggc	ccggaccg	ccggcccg	ccggccga	ccggccgg	ccggcctg
ccggcggc	ccgggagc	ccgggccg	ccgggcct	ccggggag	ccgggggc
ccggtcag	cctcagcg	cctccgcg	cctccgga	cctcgccg	cctcggag
cctcggct	cctcgggg	cctgcggg	cgaccccc	cgagcccc	cgagcctc
cgagctcg	cgccccga	cgccccgc	cgcccgcg	cgccgggc	cgccgggg
cgcgcgga	cgcgcggc	cgcggagg	cgcggccg	cgcgggca	cgcgggcg
cgcggggt	cgctcccg	cgctgagg	cggacccc	cggacccg	cggagacc
cggagccg	cggagggc	cggccccc	cggccccg	cggcccga	cggcccgc
cggcccgg	cggccctc	cggccctg	cggccgac	cggccgcg	cggcgagg
cggcgccc	cggcgccg	cggcgggc	cggctccc	cggctccg	cgggaccc
cgggagag	cgggagcc	cgggagcg	cgggcccg	cgggccgg	cgggccgt
cggggcac	cggggccg	cggggcct	cggggcgg	cgggggcc	cgggtccg
cggtccgg	ctaccccc	ctccccgg	ctcccggg	ctccgacc	ctccgagg
ctccgcgc	ctccggag	ctccgggg	ctccgtgg	ctcggccc	ctcgggac
ctcgggcc	ctctcccg	ctgaccgg	ctggcccc	gaggctcg	gagggccg
gatcccta	gccccccc	gcccccgg	gccccgtg	gcccgagt	gcccgccc
gcccgccg	gcccgcgc	gcccgcgg	gcccgcgt	gcccggcg	gcccgggc
gcccgggg	gcccgggt	gccctccg	gccgccgg	gccgcgcg	gccggccc
gcgagccc	gcgcggag	gcgcgggc	gcgcgggg	gcggaggg	gcggcccc
gcggccgc	gcggcctg	gcggctcc	gcgggccg	gcggggcg	gcgggggg
gcggtccc	gctcccgg	gctccggg	gctctcgg	ggactccc	ggagccgc
ggccagga	ggcccccg	ggcccgag	ggcccgga	ggcccggg	ggccggga
ggccgggg	ggctcccg	gggaccgc	gggagccg	gggagtcc	gggatccc
gggcccgt	gggccgag	gggccgca	gggccggc	gggcgccg	gggcgcgg
gggcgggc	gggctcgc	ggggccag	ggggccgc	ggggctcg	gggggccg
gggggcct	gggggggc	gggggggg	ggggggtt	gggggtag	gggggtcg
ggggtccg	gggtaccc	gggtcccg	gggtccga	ggtcccgt	ggtcggag
ggtctccg	gtcccgag	gtcggccg	gtgccccg	tagggatc	tcccggcc
tccgcgcg	tccgcgga	tccggagg	tccgggcc	tccgtggg	tcctggcc
tcggaccc	tcggccga	tcggccgg	tcgggccg	tcggggcg	tctccgtg
tgcccgcg	tgcggccc				

**Table 10:** Unwords for the *M. jannaschii *genome.

cgatcg	gcgcgc	gtcgac

**Table 11:** Unwords for the *T. kodakarensis *genome.

tactagta

**Table 12:** Unwords for the *M. genitalium *genome.

ccggcc	cgcgcg	ctcgga	ggccgg	tccgag

## Conclusion

Genomic unwords may not have a functional meaning, but they do have relevance in practice and in theory. When planning experiments such as large scale mutagenesis [22], a high number of markers is to be included in the inserted DNA. Such markers should be disjoint from each other and from the original genome. Given (say) 100 unwords of length 11, we can directly compose 10,000 markers of length 22 which have a guaranteed Hamming distance from the genome of at least 2. From this supply of candidates, markers can be selected according to other criteria such as melting temperature.

Unwords analysis is fast enough to be applied to the large mammalian genomes. and even to larger data sets resulting from ultra-fast sequencing projects. The fact that the genome sequence need not be kept in main memory makes the program applicable to even larger data volumes in pan- or meta-genome projects. For demonstration, we have applied our program to a recent version of the NT-database (all non-redundant GenBank+EMBL+DDBJ+PDB sequences, 21,789,632,349 bp). It requires 136 minutes and 40 MB of main memory to compute all 15,560 unwords of length 14. A further interesting application would be for genomic fragment data. In meta-genome projects based on ultrafast sequencing technology, unwords analysis may prove useful in monitoring coverage.

Unwords, by definition, always have a fixed length (say *k*) in a given genome. Longer absent words may also be of interest. They are easily determined with our program: Adding all unwords as additional sequences to the genome and re-running the program, it will produce all absent words of length *k *+ 1, since they are the unwords of the extended genome.

No evidence has been collected for selection against specific words in a genome-wide fashion. Naturally, unwords tend to have atypical CG content in the AT-rich genomes we studied (see Table 4). CpG methylation and subsequent mutation favors unwords containing CG dinucleotides, and leads to an overabundance of their mutated variants [10]. These observations suggest that length and number of unwords, and in particular their deviation from expectation in random sequences, are statistical footprints of the process of real genome evolution. Mathematical models or reconstructions of genome evolution should be tested whether they produce a similar footprint.

The program UNWORDS is available from the Bielefeld University Bioinformatics Server [23]. While online use is restricted to sequence uploads of at most 5 Mb, the UNWORDS source code is available at [24], which has no such restriction.

## Authors' contributions

RG designed and guided the study. SK provided two implementations of unword computation, one as an extension to VMATCH, and the UNWORDS program described here. JH ran the unword computations as well as all the additional analyses. All authors contributed to writing the article.
